# Editorial: Cancer evolution

**DOI:** 10.3389/fgene.2023.1187687

**Published:** 2023-04-14

**Authors:** Luca Ermini, Diego Mallo, Dimitrios Kleftogiannis, Ahmet Acar

**Affiliations:** ^1^ NORLUX NeuroOncology Laboratory, Department of Cancer Research, Luxembourg Institute of Health, Luxembourg, Luxembourg; ^2^ Arizona Cancer Evolution Center, Biodesign Institute and School of Life Sciences, Arizona State University, Tempe, AZ, United States; ^3^ Department of Informatics, Computational Biology Unit and Centre for Cancer Biomarkers, University of Bergen, Bergen, Norway; ^4^ Department of Biological Sciences, Middle East Technical University, Universiteler Mah, Ankara, Turkiye

**Keywords:** cancer evolution, evolutionary biology, somatic cell, cancer phylogenetics, tumor heterogeneity

## Introduction

Cancer is a complex disease (Donaldson et al., 2015; Yalcin et al., 2020) characterised by the breakdown of multicellular cooperation between somatic cells (Aktipis et al., 2015) that results in a set of convergent (Fortunato et al., 2017) traits known as *the hallmarks of cancer* (Hanahan, 2022). Cancer cells can proliferate extensively within a tissue, spread beyond the normal regulatory boundaries, and even colonise distant sites (Hanahan, 2022). Cancer progression is understood to be a complex Darwinian adaptive system (Greaves and Maley, 2012), with cancer cells acting as the equivalent of an asexually reproducing, unicellular quasi-species. Tumours are defined as large populations of genetically-diverse cell subpopulations competing for a limited number of nutrients while being selectively influenced by endogenous and exogenous factors. Because evolution and Darwinian selection are contingent and blind to the future, the outcome of this competition is the survival of clones well suited to flourish under specific conditions. Many clones dominant at one point in time may reach evolutionary dead ends and die out, while others may be able to persist. The level of genetic diversity found in cancer cannot be fully explained by Darwinian evolution, so non-Darwinian mechanisms have been proposed to account for cancer evolution (Vendramin et al., 2021). Neutral evolution, macroevolutionary changes, and the role of non-genetic determinants all appear to impact cancer progression significantly (Williams et al., 2016). The architecture of cancer seems sophisticated and intricate, and a thorough understanding of evolution is required for developing novel, evolutionary-informed therapeutic approaches (Gatenby and Brown, 2020). For this Research Topic, we gathered five studies covering three key aspects of cancer evolution.

### Somatic population genetics

Understanding the dynamics and possibly predicting cancer progression requires knowing how evolutionary forces alter the allele frequencies in somatic cells. Population genetic studies have generated critical insights in cancer evolution (Williams et al., 2019), including two of the articles in this Research Topic.

Luddy et al. investigated the principle of *evolutionary triage* (Gatenby et al., 2014), which connects the frequency of a gene mutation in a population with its contribution to cancer cell fitness. A mutation that improves fitness will spur cell proliferation, increasing the likelihood of its being found in a Research Topic of tumours. Instead, a mutation that does not affect fitness will only be detected at a frequency consistent with the underlying mutation rate. Luddy et al. used this framework to investigate genes associated with tumour-immune interactions in two lung adenocarcinoma cohorts with different molecular subtypes and found distinct convergent signatures of purifying selection. Targeting genes or molecular pathways under purifying selection may have therapeutic efficacy similar to targeting driver genes and may open up new therapeutic pathways (Gatenby and Brown, 2020).

Kurpas and Kimmel developed two alternative evolution models and applied them to cancer data to test if cancer cells follow Darwinian evolution. Both models describe the combined fitness effect of driver and passenger mutations in cancer cells; the only difference is how cellular fitness affects the likelihood of occupying the space of a dying cell. In one model, population fitness is constant in the absence of mutations, consistent with Darwinian selection, whereas fitness is not constant in the alternative. The two models were fitted to breast cancer samples, and it was found that the model consistent with Darwinian selection fits the data better.

### Molecular signatures

Molecular signatures can elucidate mechanisms of cancer progression and inform the clinical practice of many cancer types, perhaps most notably breast cancer (Lal et al., 2017).

Yan et al. developed a novel prognostic signature grounded on a set of necroptosis-related genes to predict breast cancer patient outcomes based on median risk scores. Patients above and below the median are classified as high-risk and low-risk, respectively. High-risk patients encounter low overall survival and worse predicted tumour, node, and metastasis stages.

Cisneros et al. studied the role of stress-induced mutagenesis (SIM) in cancer and introduced new statistical methods to identify its signature. Using somatic mutations found in tumour and normal tissue and SIM cell lines, they identified clusters of mutations consistent with stress-induced mutagenesis. These genetic clusters were less conserved in cancer, indicating a loss of regulatory control of SIM, and stress-induced mutagenesis was related to patient survival. This study shows that an evolutionary conserved adaptive mutation response, already present in bacteria, is a source of genomic instability that fuels cancer initiation, progression, and therapeutic resistance (Fitzgerald et al., 2017). This process may explain tumours’ ability to evolve under selective pressures, supporting evolutionary-based treatment strategies (Natterson-Horowitz et al., 2023).

### Integrative multi-omics

Multi-omic studies explore the interaction between multiple biological factors to provide a more holistic view of biological processes and their relative effect on an outcome (Song et al., 2020). The development of a new multi-omics tool completes this Research Topic on cancer evolution.

Huzar et al. describe MOCA, a multi-omics toolkit for comparing gene expression and genetic evolution patterns in single cells and testing their consistency. MOCA was applied to published datasets and revealed links between genetic and phenotypic changes that can aid in understanding tumorigenesis, therapy resistance, and cancer progression. A significant shift toward single-cell technologies is expected in the coming years (Rozenblatt-Rosen et al., 2020), and MOCA will enable better exploitation of these upcoming datasets.

## Conclusion

Cancer is an evolutionary process of somatic cells, and therefore the somatic evolutionary theory is critical to understanding and treating this major cause of death. The articles in this Research Topic emphasise and exemplify this fact. Evolutionary therapies are promising treatment strategies that use the most recent advances in somatic evolutionary theory to manipulate the evolution of cancer cells, controlling their growth or driving them to extinction. These advancements have the potential to improve the quality of life of cancer patients by transforming cancer into a chronic disease or, in some cases, eradicating it.

## References

[B1] Aktipis C. A. Boddy A. M. Jansen G. Hibner U. Hochberg M. E. Maley C. C. (2015). Cancer across the tree of life: Cooperation and cheating in multicellularity. Philos. Trans. R. Soc. Lond. B Biol. Sci. 370. 10.1098/rstb.2014.0219 PMC4581024 26056363

[B2] Donaldson P. Daly A. Ermini L. Bevitt D. (2015). Genetics of complex disease. Garl. Sci. 2015, 450. 10.1201/9780429258688

[B3] Fitzgerald D. M. Hastings P. J. Rosenberg S. M. (2017). Stress-induced mutagenesis: Implications in cancer and drug resistance. Annu. Rev. Cancer Biol. 1, 119–140. 10.1146/annurev-cancerbio-050216-121919 29399660 PMC5794033

[B4] Fortunato A. Boddy A. Mallo D. Aktipis A. Maley C. C. Pepper J. W. (2017). Natural selection in cancer biology: From molecular snowflakes to trait hallmarks. Cold Spring Harb. Perspect. Med. 7, a029652. 10.1101/cshperspect.a029652 28148564 PMC5287060

[B5] Gatenby R. A. Brown J. S. (2020). Integrating evolutionary dynamics into cancer therapy. Nat. Rev. Clin. Oncol. 17, 675–686. 10.1038/s41571-020-0411-1 32699310

[B6] Gatenby R. A. Cunningham J. J. Brown J. S. (2014). Evolutionary triage governs fitness in driver and passenger mutations and suggests targeting never mutations. Nat. Commun. 5, 5499. 10.1038/ncomms6499 25407411 PMC4260773

[B7] Greaves M. Maley C. C. (2012). Clonal evolution in cancer. Nature 481, 306–313. 10.1038/nature10762 22258609 PMC3367003

[B8] Hanahan D. (2022). Hallmarks of cancer: New dimensions. Cancer Discov. 12, 31–46. 10.1158/2159-8290.CD-21-1059 35022204

[B9] Lal S. McCart Reed A. E. de Luca X. M. Simpson P. T. (2017). Molecular signatures in breast cancer. Methods 131, 135–146. 10.1016/j.ymeth.2017.06.032 28669865

[B10] Natterson-Horowitz B. Aktipis A. Fox M. Gluckman P. D. Low F. M. Mace R. (2023). The future of evolutionary medicine: Sparking innovation in biomedicine and public health. Front. Sci. 1. 10.3389/fsci.2023.997136 PMC10590274 37869257

[B11] Rozenblatt-Rosen O. Regev A. Oberdoerffer P. Nawy T. Hupalowska A. Rood J. E. (2020). The human tumor atlas network: Charting tumor transitions across space and time at single-cell resolution. Cell 181, 236–249. 10.1016/j.cell.2020.03.053 32302568 PMC7376497

[B12] Song M. Greenbaum J. Luttrell J. 4thZhou W. Wu C. Shen H. (2020). A review of integrative imputation for multi-omics datasets. Front. Genet. 11, 570255. 10.3389/fgene.2020.570255 33193667 PMC7594632

[B13] Vendramin R. Litchfield K. Swanton C. (2021). Cancer evolution: Darwin and beyond. EMBO J. 40, e108389. 10.15252/embj.2021108389 34459009 PMC8441388

[B14] Williams M. J. Sottoriva A. Graham T. A. (2019). Measuring clonal evolution in cancer with genomics. Annu. Rev. Genomics Hum. Genet. 20, 309–329. 10.1146/annurev-genom-083117-021712 31059289

[B15] Williams M. J. Werner B. Barnes C. P. Graham T. A. Sottoriva A. (2016). Identification of neutral tumor evolution across cancer types. Nat. Genet. 48, 238–244. 10.1038/ng.3489 26780609 PMC4934603

[B16] Yalcin G. D. Danisik N. Baygin R. C. Acar A. (2020). Systems biology and experimental model systems of cancer. J. Pers. Med. 10, 180. 10.3390/jpm10040180 33086677 PMC7712848

